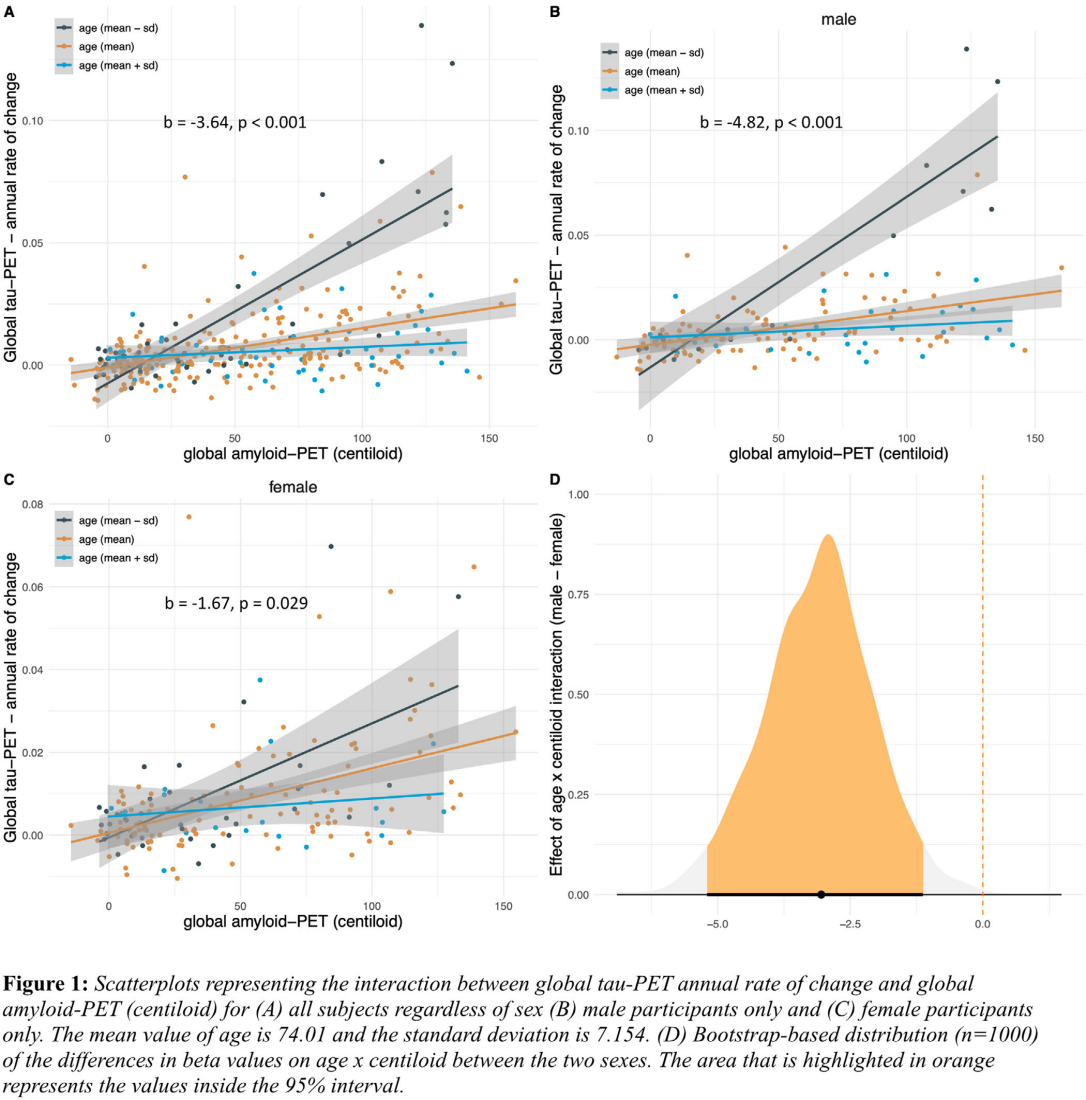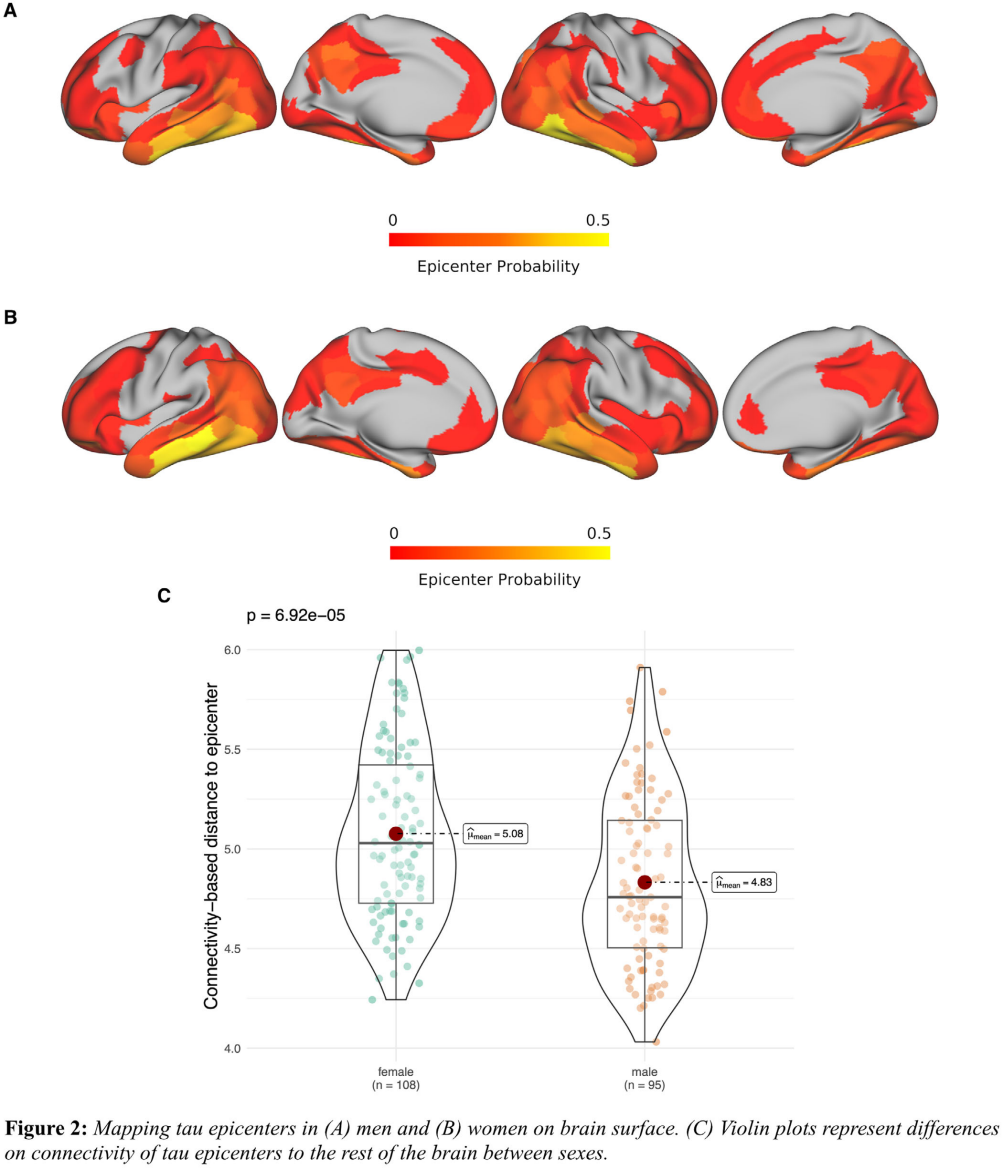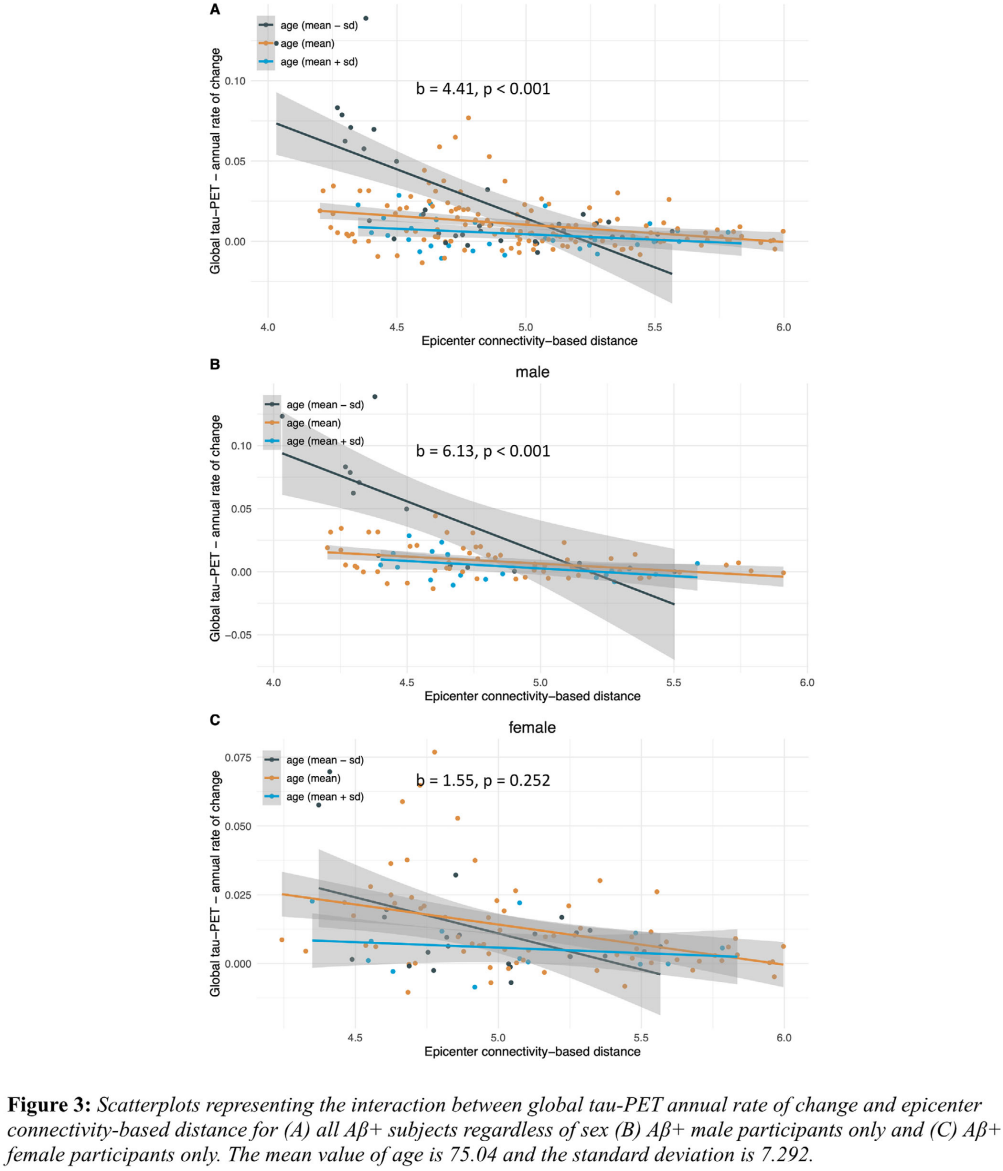# The association between the age and the rate of tau accumulation and spreading in different sexes

**DOI:** 10.1002/alz.093906

**Published:** 2025-01-09

**Authors:** Zeyu Zhu, Amir Dehsarvi, Sebastian Niclas Roemer, Anna Steward, Anna Dewenter, Mattes Gross, Fabian Wagner, Michael Ewers, Matthias Brendel, Nicolai Franzmeier

**Affiliations:** ^1^ Institute for Stroke and Dementia Research (ISD), University Hospital, LMU, Munich, Bavaria Germany; ^2^ Department of Neurology, University Hospital, LMU, Munich, Bavaria Germany; ^3^ German Center for Neurodegenerative Diseases (DZNE), Munich, Bavaria Germany; ^4^ University of Gothenburg, The Sahlgrenska Academy, Institute of Neuroscience and Physiology, Psychiatry and Neurochemistry, Gothenburg Sweden

## Abstract

**Background:**

Neuroimaging studies have revealed age and sex‐specific differences in Alzheimer’s disease (AD) trajectories. However, how age and sex modulate tau spreading remains unclear. Thus, we investigated how age and sex modulate the amyloid‐beta (Aß)‐induced accumulation and spreading of tau pathology from local epicenters across connected brain regions.

**Method:**

We included 313 ADNI participants (female/male, n=167/146), i.e. 110 cognitively normal (CN) Aß‐negative, and 203 Aß‐positive subjects across the AD spectrum (i.e. CN/MCI/Dementia, n=98/70/35) with baseline amyloid‐PET and longitudinal Flortaucipir tau‐PET. Annual tau‐PET change rates for 200 cortical regions of the Schaefer atlas were calculated. Sex‐specific resting‐state fMRI‐connectivity templates across the 200 Schaefer regions were determined in independent Aß‐negative controls (female/male, n=118/82) to determine the connectivity of tau epicenters to the rest of the brain. Using linear regression, we investigated interactions between age, sex and Aß on tau accumulation and spread, controlling for APOE4‐status and diagnosis.

**Result:**

Higher Aß (i.e. centiloid) predicted faster tau accumulation, where this association was pronounced in younger individuals (i.e. age x centiloid interaction, b=‐3.64, p<0.001, Figure 1A). This age x centiloid interaction was stronger in men (b=‐4.82, p<0.001, Figure 1B) vs. women (b=‐1.67, p=0.029, Figure 1C), suggesting that younger age promotes Aß‐related tau accumulation predominantly in men. Bootstrapping analysis further confirmed this effect (Figure 1D). In Aß+, epicenters with highest baseline tau‐PET showed a similar temporal‐lobe distribution in men and women (Figure 2A&B), yet epicenter connectivity to the rest of the brain was stronger in men vs. women (Figure 2C). Stronger connectivity of tau epicenters to the rest of the brain was linked to faster tau accumulation especially in younger Aß+ subjects (i.e. interaction age x epicenter connectivity, b= 4.41, p<0.001, Figure 3A). However, this effect was clearly driven by men (b=6.13, p<0.001, Figure 3B) and not observed when tested in women only (b=1.55, p=0.252, Figure 3C).

**Conclusion:**

Aß drives faster tau accumulation and this effect is particularly strong at younger age and even further pronounced in men, whose tau epicenters are more densely interconnected with the rest of the brain. Together, age and sex have clear modulating effects on tau spreading, and heterogeneous AD trajectories may be partly arisen due to sex‐specific differences in brain network architecture.